# Charging for the use of survey instruments on population health: the case of quality-adjusted life years

**DOI:** 10.2471/BLT.19.233239

**Published:** 2019-10-28

**Authors:** Yot Teerawattananon, Alia CG Luz, Anthony Culyer, Kalipso Chalkidou

**Affiliations:** a Health Intervention and Technology Assessment Program (HITAP), 6th floor, 6th building, Department of Health, Ministry of Public Health, Tiwanon Rd, Taladkhwan, Mueang Nonthaburi, Nonthaburi 11000, Thailand.; b University of York, Centre for Health Economics, York, England.; c MRC Centre for Global Infectious Disease Analysis, Imperial College London, London, England.

## Abstract

A trend towards charging for access to research findings, tools and databases is becoming more prominent globally. But charging for the use of research tools and databases that are vital to research supporting national and international policy development might be unjustified. Financial barriers to accessing these tools and databases disproportionately affect low- and middle-income countries, who may have greater need for information that fuels research in their areas of concern. However, changing this trend is potentially possible. One example is the experience with the EuroQol-five-dimensional questionnaire (EQ-5D), a generic measure of health status used in economic evaluations for resource allocation decisions. Increasingly, governments and health-care providers are using the EQ-5D tool in patient-reported outcome measures to monitor quality of health-care provision, diagnose and track disease progression, and involve patients in their health care. The EuroQol Group, which owns the intellectual property rights to the EQ-5D, recently terminated their policy of charging for noncommercial, nonresearch uses of the tool. We share a brief history of this development and examine these charging policies in the context of the EQ-5D’s use in national health-care research and policies, reflecting the trends and developments in the use of survey instruments on population health.

## Résumé

On observe à l'échelle mondiale une tendance de plus en plus marquée à la tarification de l'accès aux résultats, aux outils et aux bases de données de recherche. Cependant, la tarification de l'utilisation d'outils et de bases de données essentiels aux recherches appuyant l'élaboration de politiques nationales et internationales n'est pas toujours justifiée. Les obstacles financiers qui entravent l'accès à ces outils et bases de données affectent de manière disproportionnée les pays à revenu faible et intermédiaire, qui peuvent avoir davantage besoin d'informations pour alimenter des recherches sur leurs sujets de préoccupation. Il est toutefois possible d'infléchir cette tendance. Le questionnaire EuroQol-five-dimensions (EQ-5D), une mesure générale de l'état de santé utilisée dans les évaluations économiques pour les décisions relatives à l'affectation des ressources, constitue un exemple. Les gouvernements et les prestataires de soins de santé utilisent de plus en plus l'outil EQ-5D dans les mesures des résultats notifiés par les patients pour surveiller la qualité de la prestation des soins de santé, diagnostiquer et suivre l'évolution d'une maladie, et associer les patients à leurs soins de santé. Le groupe EuroQol, qui détient les droits de propriété intellectuelle relatifs à l'EQ-5D, a récemment mis fin à sa politique de tarification pour l'utilisation de l'outil à des fins non commerciales et dans un contexte autre que celui de la recherche. Nous retraçons brièvement ce cheminement et examinons ces politiques de tarification dans le contexte de l'utilisation de l'EQ-5D dans les recherches et politiques nationales sur les soins de santé, en rendant compte de l'évolution de l'utilisation du matériel d'enquête sur la santé de la population.

## Resumen

La tendencia a cobrar por el acceso a los resultados de las investigaciones, las herramientas y las bases de datos está cobrando cada vez más importancia en todo el mundo. Sin embargo, puede que no esté justificado cobrar por el uso de herramientas de investigación y bases de datos que son vitales para la investigación en apoyo del desarrollo de políticas nacionales e internacionales. Las barreras financieras para acceder a estas herramientas y bases de datos afectan desproporcionadamente a los países de ingresos bajos y medios, que pueden tener una mayor necesidad de información que impulse la investigación en sus áreas de interés. Sin embargo, es posible cambiar esta tendencia. Un ejemplo es la experiencia con el cuestionario de cinco dimensiones EuroQol (EQ-5D), una medida genérica del estado de salud utilizada en las evaluaciones económicas para las decisiones de asignación de recursos. Cada vez más, los gobiernos y los proveedores de la atención de la salud están utilizando la herramienta EQ-5D en las medidas de resultado informadas por el paciente para supervisar la calidad de la prestación de la atención de la salud, diagnosticar y hacer un seguimiento de la progresión de la enfermedad e involucrar a los pacientes en su cuidado de la salud. El Grupo EuroQol, propietario de los derechos de propiedad intelectual de EQ-5D, puso fin recientemente a su política de cobrar por los usos no comerciales y no relacionados con la investigación de la herramienta. En este artículo se presenta una breve historia de este desarrollo y se examinan estas políticas de cobro en el contexto del uso de EQ-5D en la investigación y las políticas nacionales de atención de la salud, reflejando las tendencias y los avances en el uso de los instrumentos de las encuestas sobre la salud de la población.

## ملخص

أصبح الاتجاه نحو فرض رسوم مقابل الاطلاع على نتائج الأبحاث والأدوات وقواعد البيانات، أكثر وضوحاً على مستوى العالم. إلا أن فرض رسوم على استخدام أدوات البحث وقواعد البيانات التي تعد ضرورية للبحث الذي يدعم تطوير السياسات الوطنية والدولية، أمراً غير مبرر. إن العقبات المالية التي تعوق الاطلاع على هذه الأدوات وقواعد البيانات تؤثر بشكل غير متناسب على البلدان ذات الدخل المنخفض والمتوسط، والتي قد تكون بحاجة أكبر إلى المعلومات التي تغذي الأبحاث في مجالات اهتمامها. إلا أن تغيير هذا الاتجاه أمر ممكن ومحتمل. ومثال على ذلك تجربة استبيان EuroQol خماسي الأبعاد (EQ-5D)، وهو مقياس عام للحالة الصحية يتم استخدامه في التقييمات الاقتصادية لقرارات تخصيص الموارد. تستخدم الحكومات وجهات تقديم الرعاية الصحية على نحو متزايد أداة EQ-5D في مقاييس النتائج التي يبلغ عنها المريض، لمراقبة جودة توفير الرعاية الصحية، وتشخيص وتتبع تطور المرض، مع إشراك المرضى في الرعاية الصحية المقدمة لهم. قامت مجموعة EuroQol Group، التي تمتلك حقوق الملكية الفكرية لـ EQ-5D، مؤخراً بإنهاء سياستها الخاصة بفرض رسوم على استخدامات الأداة لأغراض غير تجارية وغير بحثية. نحن نقوم بعرض تاريخ موجز لهذا التطور، ونختبر تلك السياسات الخاصة بفرض الرسوم في سياق استخدام EQ-5D في الأبحاث والسياسات الوطنية للرعاية الصحية، مما يعكس الاتجاهات والتطورات في استخدام أدوات المسح الإحصائي على صحة السكان.

## 摘要

在全球范围内，要求付费以获取研究成果、工具和数据库的现象逐渐成为一种重要趋势。但是，使用对支持国家和国际政策发展的研究至关重要的研究工具和数据库时，相关收费并不合理。为访问这些工具和数据库设置的资金障碍对中低收入国家的影响尤为严重，这是因为中低收入国家可能需要更多信息以推动其相关领域的研究发展。但是，或许可以改变这种趋势。以使用 EuroQol 五维问卷（EQ-5D，在资源分配决策相关经济评估方面使用的通用健康状态测量量表）的经验为例。越来越多的政府部门和医疗保健提供者在患者报告结局测量方面使用 EQ-5D 工具，以监控医疗保健质量、诊断和跟踪疾病进展并吸引患者参与其医疗保健项目。拥有 EQ-5D 的知识产权的欧洲生存质量学会最近终止了一项政策，即其针对出于非商业和非研究目的使用该工具的情况实施的收费政策。我们简单分享了其发展史，并基于 EQ-5D 在国家医疗保健研究和政策领域的使用情况，研究了这些收费政策，以反映使用人口健康调查工具的趋势和发展。

## Резюме

Тенденция к начислению платы за доступ к результатам, инструментам и базам данных исследований приобретает ярко выраженный характер по всему миру. Но взимание платы за использование инструментов и баз данных исследований, совершенно необходимых для поддержки развития национальных и международных политик, может быть необоснованным. Финансовые барьеры к использованию этих инструментов и баз данных могут непропорционально сильно сказаться на странах со средним и низким уровнем дохода, которые могут испытывать большую потребность в информации, поддерживающей проведение исследований в проблемных для данных стран областях. Однако существует потенциальная возможность изменить такую тенденцию. В качестве одного из примеров можно привести опросник из пяти вопросов EuroQol (EQ-5D) — общий показатель состояния здоровья, используемый при экономической оценке для принятия решений о распределении ресурсов. Правительства стран и медицинские учреждения все чаще используют инструмент EQ-5D для измерения результатов, сообщаемых пациентами, с целью контроля качества оказания медицинской помощи, диагностики и отслеживания течения заболевания, а также вовлечения пациентов в процесс медицинского обслуживания. Компания EuroQol Group, которая владеет правами интеллектуальной собственности на EQ-5D, недавно отказалась от политики начисления платы за некоммерческое использование инструмента, не связанное с проведением исследований. Авторы вкратце рассказывают о развитии данной ситуации и рассматривают политики относительно начисления платы в контексте использования EQ-5D в национальных исследованиях и разработке политик в области здравоохранения, отражающих тенденции и изменения в использовании средств исследования здоровья населения.

## Introduction

Cross-country and inter-institutional research collaborations are progressive developments in an increasingly globalized and interdependent world. However, the improvements in governance and policies surrounding distribution and use of research articles, databases and data collection tools have lagged behind. The need for socially responsible licensing policies for access to data and tools highlights the growing commercialization of the products of research. We can contrast this with universities’ and public health organizations’ goals of contributing to the public interest. Commercialization often leads to patents and intellectual property management becoming restrictive for research and product use in low- and middle-income countries. For example, a university-developed, but privately funded research on a drug or formulation may result in a patent that belongs to the private entity; without appropriate licensing strategies, the private entity may register the drug in many countries, which could result in limited access to the drug in resource-constrained settings. Research may also be concentrated on areas that are more beneficial to funders, as opposed to areas that would address issues for disadvantaged communities or settings.^1^ Furthermore, paywalls, such as charges for accessing journal articles, can hinder the flow of scientific information and developments that rely and build on previous research.^2^ A worrying trend is organizations charging for access to research tools and databases that are vital to research supporting national and international policy development.

Research consortia in the United Kingdom of Great Britain and Northern Ireland and the United States of America routinely restrict access to their economic models or global health estimates.^3^ The website of United States National Guideline Clearinghouse, which housed more than 2000 easily and freely accessible guidelines for evidence-based health care, was taken down in July 2018 due to government budget cuts.^4^ The Cochrane Library, which is a repository of high-quality research to support health care decision-making globally, allows only one-time access before requiring payment, even for researchers from low- and middle-income countries.^5^ These research tools and databases are becoming increasingly hard to access. Barriers to information access are especially problematic for governments and researchers in low- and middle-income countries, who often contribute their own resources towards further development of these products. Given the importance of standard tools for supporting country and global priority-setting, research and development, it is important to examine barriers to their access.

One example of this issue is the case of the EuroQol-five-dimensional questionnaire (EQ-5D; EuroQol Group, Rotterdam, Netherlands), a tool for measuring health status. EuroQol Group is managed by the not-for-profit EuroQol Research Foundation, which owns the intellectual property rights to EQ-5D in Europe, North America and other parts of the world. In November 2018, a lawyer from the Foundation approached a researcher from the Thai Health Intervention and Technology Assessment Program to provide evidence of use of the EQ-5D tool in Thailand as support for registering the tool as a trademark. After a few exchanges, it became clear that EuroQol aimed to register the EQ-5D under a new policy that could result in potentially charging users of the tool in Thailand. Arguably, the charge was reasonable for commercial use of EQ-5D, but this new policy, dated June 2018, meant that noncommercial, nonresearch use was also to be explicitly charged.^6^ All countries, even low-income countries, would be asked to pay this charge. Following complaints by the Thai Health Intervention and Technology Assessment Program, EuroQol agreed to reconsider the policy and, in January 2019, reversed their decision.^7^^,^^8^

An important noncommercial, nonresearch use of the EQ-5D is the routine collection of patient-reported outcome measures in clinical settings. Such data form part of many health systems’ monitoring efforts to improve service quality and inform patient choice of providers. In this paper we share a brief history of licensing for the EQ-5D with the aim of informing the global health community and encouraging discussion about the development and management of similar research tools. The paper is also a call for more sharing of research tools such as executable models and databases as well as guidelines and best practice norms that have the potential to inform global and national health policies.

## EQ-5D and the global community

The EQ-5D tool is a generic measure of health status within five dimensions (mobility, self-care, usual activities, pain or discomfort, anxiety or depression) on three levels (no problems, some problems, extreme problems). The tool’s origins lie in academic papers published in the 1970s and 1980s.^9^^–^^13^ EuroQol developed the tool in the 1980s to measure, value and compare health status across disease areas, primarily with the aim of using the results to inform resource allocation decisions. EuroQol’s objectives were to develop a standardized instrument for measuring health-related quality of life, which had dimensions relevant to a broad range of patients, as well as to the general population and, which would be simple and easy to complete.^14^ The reliability and validity of the tool was tested on a variety of populations and patients, and research continues on issues, such as the effect of the duration of the health states on patients’ self-reported values and other considerations, for example the use of the tool in large-scale health-system applications. EuroQol has also published EQ-5D-5L, a more sensitive tool that includes more levels (no problems, slight problems, moderate problems, severe problems, extreme problems) and EQ-5D-Y, a tool for measuring children’s and adolescents’ health status.^14^

The EQ-5D is the most frequently used tool for generating quality-adjusted life years values. These values are applied as health outcomes in economic evaluations, a type of health technology assessment. Use of the tool grew markedly with the increasing application of health technology assessment for decision-making through the National Institute for Health and Care Excellence in the United Kingdom and similar institutions in other countries. Pharmaceutical companies included EQ-5D in their health technology assessment submissions, paying fees for its use and providing EuroQol with a revenue source. This revenue allowed EuroQol to have formalized legal arrangements, establish the EuroQol Group and Foundation and to create a business model to facilitate not-for-profit research activities. 

All users are now required to register the instrument (copyright belongs to the EuroQol Group). Only commercial, for-profit users were charged. Over time, group membership became an international network. The EuroQol Foundation has since developed procedures for ensuring that cultural adaptations of the tool retain the intended meaning of the original and a protocol for assigning valuations to ensure standardization.^11^^,^^14^ The protocol is a standard method to assess health state preferences, ensuring that the valuations remain consistent across countries with different cultures and social and economic status. 

The Foundation now provides leadership in the development of instruments for describing and valuing health, promoting the use of these tools, fostering support for the international community of researchers developing these tools and ensuring the proper use of the tools in the various contexts in which they are applied. The ease and simplicity of use of the EQ-5D have allowed it to be incorporated in clinical trials, observation studies, population health surveys and patient-reported outcome measures.^11^^,^^14^

EuroQol has promoted and supported EQ-5D use and development for research in many countries through educational and uptake initiatives. Similarly, these efforts received support from the research community globally in testing and developing local values to allow translation from EQ-5D scores to health utilities that are used in economic evaluations.^14^ For example, in 2004, the Thai Health Intervention and Technology Assessment Program used public resources to fully fund a household survey for assessing the Thai valuation of EQ-5D-3L.^15^ In 2007, the Thai health technology assessment guideline endorsed the use of EQ-5D as the preferred health utility measure for economic evaluations conducted for allocating health resources by the Thai government,^16^ one of the first low- and middle-income countries to do so. The second household survey to assess the Thai value set of EQ-5D-5L was also partially supported through Thai government funds.^17^

It is clear that, from the very beginning and throughout its subsequent development, the EQ-5D has been created and extended by researchers employed in public institutes, supported by public research funding and used in the formation of public policy.

## Are charges appropriate?

There can be advantages to charging for use of the EQ-5D. Charging enables the monitoring of the tool’s use and so maintains consistency in its meaning and use. Charges can also be used to build up reserves for supporting further research, developing the tool and promoting its use in research and clinical practice. Furthermore, charges fund EuroQol staff who receive applications for translation and use, respond in a timely manner to questions and organize conferences and meetings with various stakeholders to learn about the tool and its applicability in diverse settings.^14^ The staff members also ensure that an appropriate version of the tool is used that fits the research goals of each applicant.^11^ More countries are now inviting pharmaceutical companies to submit or conduct studies that require the use of the EQ-5D, which means there is greater potential for income generation in that area.

We believe, however, that charging should only be for commercial use of the tool. Noncommercial use of EQ-5D should remain free of charge and it is encouraging that the foundation reconsidered its charging policy.^6^^,^^8^ Since the tool was an outcome measure developed in universities for public policy purposes, it is inappropriate for those same universities and similar non-profit public agencies to be charged for its use. EQ-5D increases in value with continued use by governments and academics.^18^^–^^22^ This development might not have occurred if the charging policy had been outlined beforehand, because the process for the tool’s development has involved the sharing of resources supported by governments and many academics.

Patient-reported outcome measures are an important component of policy assessment and review. As the EQ-5D is a simple tool that is easy to complete, it is a good measure of patient-reported outcome measures with an accepted role in clinical settings for diagnosing and monitoring disease progression and patients’ health status; for facilitating communication and shared decision-making with patients; and for gathering data on the effects of interventions. Patient-reported outcome measures are reliable predictors of disease progression to complement traditional indicators (such as tumour markers or tumour response)^23^^,^^24^ and help doctors, governments and relevant stakeholders to meet the demands and needs of their patients more effectively. Such measures are especially needed by countries and health systems in their journey towards universal health coverage.

Many clinics, hospitals and government health systems in low-income countries would likely be unable to afford the user charges. Even middle-income countries that could theoretically afford to pay charges may have more pressing demands on their health research budgets. The United Kingdom currently uses the EQ-5D tool for its national programme of patient-reported outcome measures,^25^ with many countries following suit. Charging for use of the tool could eventually disincentivize its use. Governments, particularly in low- and middle-income countries with greater resource constraints, could disinvest or commit fewer resources to tools with such barriers, perhaps jeopardizing efforts to develop the EQ-5D in countries that could benefit most from its use. The loss of investment in research development needs to be balanced against the revenue gains from charging.

**Fig. 1:**
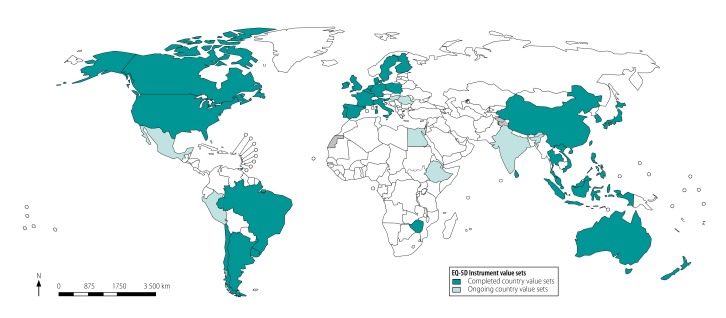
Countries with value sets for health status derived using the EuroQol-five-dimensional questionnaire

Finally, the measurement of quality-adjusted life years has been promoted globally and endorsed by health technology assessment agencies, such as the National Institute for Health and Care Excellence, National Institute for Health Research and Thai Health Intervention and Technology Assessment Program.^26^^–^^28^ The understanding among agencies was that the tools to measure quality-adjusted life years would be freely accessible. EuroQol is laudable for providing the questionnaire free of charge for such purposes. This practice contrasts with the health utilities index of Health Utilities Inc.^29^ or the six-dimensional health state short form (SF-6D) of the University of Sheffield,^30^ which have charges or financial barriers for noncommercial research use. The SF-6D, for example, allows a one-study use for non-profit and research organizations and charges subsequent studies. As in Thailand, other low- and middle-income countries are investing in the EQ-5D value sets for this reason (Fig. 1). However, changing the policy sets a precedent for future charges for other EQ-5D uses that could restrict the use of the quality-adjusted life years measure on a global level.

EQ-5D: EuroQol-five-dimensional questionnaire.

Note: The EQ-5D (EuroQol Group, Rotterdam, Netherlands) is a standardized instrument for measuring health status.^12^,^31^ The 2003 value set for Europe was derived from six countries which are highlighted in the map. The value set may have been used in other European countries.^32^

Data sources: EuroQol.^33^,^34^

## Policy questions

There are several policy-relevant questions we can pose to research agencies who are developing tools for global use when considering sustainable models of income generation. First, is it justifiable to charge for the tools needed to measure key global health indicators? Second, is it justifiable to differentiate between commercial and noncommercial uses of the tools and charge only for the former? Third, is it justifiable to differentiate between research and nonresearch uses of the tools and charge only for the latter? Fourth, do not-for-profit groups need top-up funding to supplement the revenues which they currently generate? Fifth, are there any circumstances in which it would be acceptable to charge noncommercial research users for use of these tools? Sixth, what are the best methods for informing users and keeping them up-to-date with policy changes regarding charges for use of these tools? Finally, is there a consensus concerning the answers to these questions?

## Ensuring information and research accessibility

We need to look at how to sustain high-quality research efforts while ensuring accessibility to the products and tools of such research. EuroQol’s change in policy is part of a pushback against barriers to information access, especially concerning information generated by publicly funded research. These responses include initiatives such as the Tufts Cost-effectiveness Analysis Registry and the F1000Research database that invite researchers to upload their models and papers to be freely available; the Bill and Melinda Gates Foundation and the Wellcome Trust adopting open-access policies to ensure that their funded research is accessible; and calls to allow transparency through the All Trials Campaign, an initiative that aims to have all results and methods from previous and current clinical trials registered and reported.^35^^–^^39^

Another promising initiative is socially responsible licensing, which allows licensing of technologies and medicines for defined populations to provide differential access or prices compared with regular licensing policies.^40^ Socially responsible licensing includes different licensing strategies with the end goal of safeguarding access. For example, universities in the United States of America have begun implementing these licences by defining the contractual language to ensure royalty-free or reduced fees for specific purposes or for use by low- and middle-income countries or humanitarian groups. The initiative has generated even more research. A positive development from these policies was the stimulation of the support and investment from licensees and philanthropic organizations to universities.^40^^,^^41^ Socially responsible licensing has the potential to reduce barriers to information access and research development for low- and middle-income countries, while still ensuring the sustainability, quality and development of global research and tools.
